# Use of *Bacillus velezensis* JNS-1 from vermicompost against strawberry root rot

**DOI:** 10.3389/fmicb.2025.1566949

**Published:** 2025-04-23

**Authors:** Lili Jiang, Haohao Yan, Yingzi Yin, Ran Dong, Chong Wu

**Affiliations:** ^1^Shandong Institute of Pomology, Tai’an, China; ^2^Department of Plant Protection, Shandong Agricultural University, Tai’an, China

**Keywords:** *Bacillus velezensis*, root rot, biological control, vermicompost, fungal disease

## Abstract

China is the biggest producer of strawberry and has the largest strawberry cultivation area worldwide. This study adopted an experimental method to explore the effect of vermicompost addition on the yield and quality of strawberries and to investigate the influence of the composition and activity of microbial communities in soilless cultivation systems. The addition of vermicompost significantly increased the activity of three soil enzymes, namely cellulase, urease, and acid phosphatase. Furthermore, it significantly increased the nitrogen content of the strawberry-planted substrate, through NO_3_-N and alkaline-N contents and organic matter, as well as the available-K content and cation exchange capacity, but with no significant effect on the available-P content of the substrate. Vermicompost addition effectively improved the growth of strawberry plants by transforming microbial diversity and increasing the level of beneficial microbial transcription. The average plant yield of the first-crop fruit of vermicompost-treated plants was significantly higher than that of the control. Furthermore, a biocontrol strain named JNS-1 was isolated from the vermicompost, which showed a good direct inhibitory effect against the mycelial growth of *Fusarium oxysporum and Neopestalotiopsis* spp. According to its morphology and 16S rRNA gene sequences, JNS-1 strain was identified as *Bacillus velezensis*. In greenhouse pot experiments and field trials, *B. velezensis* JNS-1 was found to be effective in controlling strawberry root rot disease. This study focuses on using *B. velezensis* JNS-1 to enhance the health and quality of strawberries and providing a theoretical basis for the use of *B. velezensis* JNS-1 in strawberry fields.

## 1 Introduction

Strawberry (*Fragaria* × *ananassa* Duch.) is a globally cultivated small fruit with significant economic and nutritional value, but with a brief history of less than 300 years (Fan and Whitaker, 2024; Fang et al., 2024; Zhang et al., 2025). It can be consumed in the fresh form (Liu et al., 2021) or canned or processed into wine, vinegar, preservatives, and other leisure food favored by the majority of consumers (Hernández-Martínez et al., 2023; Liu et al., 2023). Currently, China is the largest producer of strawberries in the world, with a plantation area of 1.3 × 10^5^ ha and an annual production of 2.8 × 10^5^ t (Sun et al., 2021; Li et al., 2023). Recently, healthy production of strawberries has attracted increased attention from many scientific researchers (Jiang et al., 2022, 2024a).

Strawberry production is significantly affected by environmental factors (Tang et al., 2020). In addition, relying on soil cultivation and continuous cultivation of strawberries can lead to various emerging plant diseases and cause significant losses to the yield and quality of strawberries (Jiang et al., 2024b; Wang et al., 2023). Thus, soilless cultivation systems have been used as an intensive and sustainable method for planting strawberries in response to problems such as the reduction in cultivated land, a lack of water resources, and poor soil quality (De Nardi et al., 2024). One such method uses coconut bran, which is an agricultural waste product (Lin et al., 2023). In recent years, due to its good physical properties, coconut bran has been used as the substrate in soilless cultivation systems. Vermicompost, the by-product obtained from the disposal of biowastes by earthworm breeding, is rich in key substances required for crop growth, such as plant growth regulators and humic acid salts, and improves soil physicochemical properties (Wang et al., 2017; Guo et al., 2023). Meanwhile, vermicompost can optimize the composition of microbial communities involved in soil productivity, which can further enhance the secondary productivity of the soil, inhibit plant diseases, and promote plant growth (Guo et al., 2023; Yatoo et al., 2021). To date, only a few reports have evaluated whether vermicompost can be applied to soilless cultivation systems and its impact on strawberry pots and yield and whether vermicompost addition affects the composition and activity of microbial communities in China (Cabilovski et al., 2023; De Nardi et al., 2024). Therefore, the present study focuses on exploring the diversity and characteristics of beneficial microorganisms present in vermicompost.

*Fusarium oxysporum* and *Neopestalotiopsis* sp. can cause strawberry root rot, which are pathogenic bacteria that primarily invade plants continuously from the root epidermal cells (Gordon et al., 2019; Baggio et al., 2023). Once they reach the vascular bundle, the pathogenic bacteria extend along the vascular bundle, break the duct function, and create a dead plant, which can be harmful to the entire growth period of the plant. Therefore, it is difficult to control straw root rot caused by *F. oxysporum.*

Chemical measures to control root rot diseases in strawberries are easy to implement, but they cause pollution (Bi et al., 2024). Hence, it is necessary to find effective biocontrol strains to prevent and control these diseases. Vermicompost can increase crop growth and yield and suppress diseases and pests sustainably, without affecting human health and the environment (Yatoo et al., 2021; Cabilovski et al., 2023; De Nardi et al., 2024). The present study adopted an experimental method to explore the effect of adding vermicompost on the yield and quality of strawberries and the influence of the composition and activity of microbial communities in soilless cultivation systems. The trials were carried out in soilless systems and then again in the field using field soils, and this study did not imply shifting from the soil cultivation system to the soilless cultivation system. JNS-1, a biochemical strain, was isolated from the vermicompost and identified as *Bacillus velezensis.* This study provides a theoretical basis for the control of root rot diseases using *B. velezensis* in strawberry.

## 2 Materials and methods

### 2.1 Field experiment design

Two treatments were carried out in this study. Treatment one included the use of coconut bran, provided by Xiamen Huanshi Import and Export Trading Co., Ltd., as the substrate. After washing, the electrical conductivity (EC) of the coconut bran was 0.7–0.9 ms/cm. In treatment two, the substrate comprised coconut bran and vermicompost in a ratio of 16:1, and the vermicompost was fermented using rice husk and cow dung as carriers. Both treatments were repeated three times, and each planting area was considered one plot with a length of 10 m, depth of 35 cm, and width of 30 cm. On 11 September 2021, “Zhangji” strawberry cultivar seedlings (provided by Kunming Kusen agricultural development Co., Ltd.) with three leaves and one bud were transplanted. A “W” type double-row planting, with a row spacing of 20 cm × 20 cm, was followed, and approximately 100 healthy “Zhangji” seedlings were transplanted into each plot (N36°05′53.31″, E116°57′53.16″). To enhance the reproducibility of the study, the agricultural procedures of the two treatments were kept consistent. After sampling for measuring substrate nutrients, a large amount of elemental water-soluble fertilizer containing N, P, and K content of 20% each was applied. Every 15–20 days, 50 g of the fertilizer was wash-applied per implant until the end of the harvesting period.

### 2.2 Strawberry plant growth

Forty days after the strawberry seedlings were transplanted, 15 strawberry plants were randomly selected from each plot. Their height and canopy were measured using a ruler, while their relative chlorophyll content and leaf area of the third developed leaf were determined using a SPAD-502 handheld chlorophyll analyzer (SPAD-502 +, Konica Minolta, Inc.) and a YMJ leaf area analyzer (CI-202, CID Bio-Science, Inc.), respectively. In addition, 30 days (growth period) and 120 days (fruiting period) after transplantation, three random strawberry plants from each plot were uprooted and rinsed thoroughly, and their surface was dried out. The aboveground and belowground parts of strawberry plants were separated and weighed separately to obtain measurements of their fresh weight (Shrestha et al., 2024).

### 2.3 Enzyme activity and elemental determination

The activities of soil enzymes, including cellulase, urease, and phosphatase, as well as plant nutrient contents, were measured following the established protocols (Li et al., 2020). Cellulase activity was quantified using the Grice Soil Cellulase Kit (G0308F), Suzhou, China. Urease activity was assessed using the conductivity method, whereas acid phosphatase activity was determined using the acetate method. In addition, nitrate content was analyzed via ultraviolet spectrophotometry. Alkali-hydrolyzable nitrogen content was measured using the alkali diffusion method. Furthermore, available-P content was determined through sodium bicarbonate extraction followed by the molybdenum–antimony anti-colorimetric method, and available-K content was assessed using ammonium acetate extraction and flame spectrophotometry. Organic matter content was quantified using the hydrated potassium dichromate oxidation colorimetric method. Moreover, cation exchange capacity (CEC) was determined using the single balance method.

### 2.4 Substrate sampling and transcriptome analysis

Substrate sampling was carried out 40 days after strawberry seedling transplantation. First, 15 sampling points of approximately 5 cm from strawberry plants were randomly selected in each plot. After removing the surface matrix, the substrate was collected from a depth of 5–15 cm, thoroughly mixed, sieved through a 2 mm mesh, and divided into two parts. Part 1 was stored at 4°C to determine soil enzyme activity and physicochemical properties. Part 2 was freeze-dried with liquid nitrogen and stored in dry ice for high-throughput sequencing to analyze fungal and bacterial diversity. At the peak fruiting stage of 4 months after transplanting, substrate samples were taken again using the same method. The samples were thoroughly mixed, sieved through a 2 mm mesh, and divided into two parts, both of which were freeze-dried with liquid nitrogen. While part one was used for high-throughput sequencing to analyze fungal and bacterial diversity, part two was used for the transcriptome analysis of microbial substances. Sequencing technology was provided by Shanghai Lingen Biotechnology Co., Ltd.

### 2.5 Strawberry fruit test and yield test

At the mature stage of the first fruit (after 90–120 days), 15 near-mature fruit were randomly selected from individual plants of each plot, and single-fruit weight and soluble solid content were measured (Chiomento et al., 2021; Drobek et al., 2024). The fruit from each plot were harvested at maturity, and the cumulative yield was calculated. To facilitate operation, the area of the aisle was kept relatively large. The average yield of each plant was calculated based on 100 plants in each plot, and the equivalent yield was calculated based on 8,000 plants per 666.7 m^2^.

### 2.6 Selected bacterial isolates from vermicompost

The sample of vermicompost was collected, air-dried, and sifted using a 2 mm mesh. Then, 5.0 g of the sample was weighed and placed in a 100 mL triangle bottle, 45 mL of sterile water was added, and the bottle was swirled and oscillated for 10 min to obtain a 10^−1^ sample suspension. Sample suspensions with concentrations of 10^−2^, 10^−3^, 10^−4^, 10^−5^, and 10^−6^ were obtained by gradient dilution in sequence. The suspensions of 100 μL with a concentration of 10^−4^, 10^−5^, and 10^−6^ were placed on Luria–Bertani (LB) medium, coated evenly, and cultured in the dark at 33°C for 2 days. Single colonies were selected and cultured on a new LB medium plate for isolation and purification, and *Fusarium oxysporum* and *Neopestalotiopsis* sp. were used for the fungi antifungal test (Cheng et al., 2019; Wang et al., 2024). The two pathogens were provided by the provincial Key Laboratory of Agricultural Microbiology, Shandong Agricultural University.

### 2.7 Identification of the antifungal bacterial strain JNS-1

The bacterial strain that had the best inhibitory effect (Wang et al., 2024) on *F. oxysporum* was selected from 15 antagonistic strains with potential biocontrol properties, and this strain was named JNS-1. The identity of the strain was determined based on multiple criteria, such as physiological and biochemical indicators according to 16S rRNA using primers 1492R/27F (1492R: 5’-GGTTACCTTGTTACGACTT-3′; 27F: 5’-AGAGTTGATCCTGGCTCAG-3′) (Wang et al., 2024). Strain morphology was observed using the LB medium in combination with a scanning electron microscope. The phylogenetic tree was constructed using the maximum likelihood method based on 16S rRNA regions performed in MEGA 7.0.

### 2.8 Evaluation of the control effect of strain JNS-1 against strawberry root rot in the greenhouse pot experiment

Strawberry seedlings of the “Zhangji” variety with three leaves and one core stage were transplanted separately in a plastic pot of 15 cm diameter with a special substrate for strawberries. The pots were further inoculated with conidial suspension (1 × 10^6^ spores/ml) of each pathogen, which was thoroughly mixed into the soil. For the treatment group that received liquid bactericide, 10 mL and 20 mL of the bactericide solution were injected into the rhizosphere of each strawberry plant, respectively, whereas, in the solid bactericide treatment group, 10 g and 20 g of the solid bactericide were applied to the rhizosphere of each plant, respectively. The control group remained untreated. Each treatment was conducted in triplicate, with 10 pots per replicate. The incidence of root rot disease was evaluated 15 and 30 days after treatment under standard cultivation conditions, and the relative control efficiency was subsequently calculated (Yang et al., 2024).

### 2.9 Evaluation of the control effect of strain JNS-1 against strawberry root rot in field trials

In the strawberry field where the “Zhangji” cultivar had been cultivated for 5 consecutive years, a severe incidence of root rot was observed. In the liquid inoculant treatment group, 10 mL and 20 mL of the liquid inoculant were poured into the rhizosphere of each strawberry plant, respectively, whereas in the solid inoculant treatment group, 10 g and 20 g of the solid inoculant were applied to the rhizosphere of each strawberry plant, respectively. Untreated strawberry seedlings were used as controls. Each treatment was carried out in triplicate, the study area was 10 m^2^, and approximately 120 strawberry seedlings were planted. The incidence of strawberry root rot disease was investigated after 15 and 30 days, and the relative control efficiency was calculated (Yang et al., 2024).

### 2.10 Data analysis

Statistical analysis was conducted using SPSS 26.0 (IBM Corporation, Armonk, NY, United States). The Shapiro–Wilk test was used to evaluate the normality of the data, and the result showed that a *p*-value of greater than 0.05 indicates that the data matched the normal distribution. Independent samples *t*-tests were performed to compare the differences between treatments.

## 3 Results

### 3.1 Effects of vermicompost application on soil enzyme activities of strawberry-planted substrate

**Figure 1:**
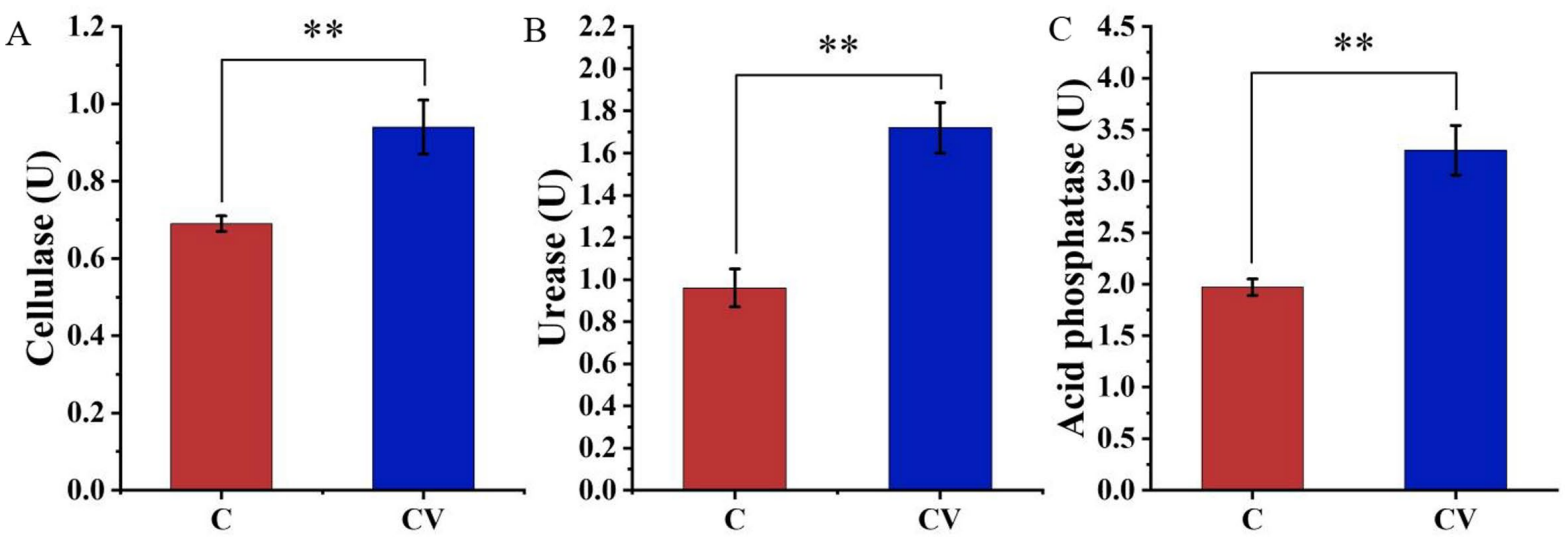
Effects of coconut-coir-only (treatment C) and coconut coir + vermicompost (treatment CV) treatments on the activity of three soil enzymes: **(A)** cellulase; **(B)** urease; **(C)** acid phosphatase. Data are mean ± SD (*n* = 15). * *p* < 0.05, ** *p* < 0.01, *** *p* < 0.001.

The addition of vermicompost to coconut coir significantly increased the activity of three soil enzymes, namely cellulase, urease, and acid phosphatase, whose activities were 0.94, 1.72, and 3.30 U, respectively, which were significantly higher than those in the coconut-coir-only treatment (0.69, 0.96, and 1.97 U, respectively) (*p* < 0.01) (Figure 1).

### 3.2 Effects of vermicompost addition on nutrient contents of strawberry-planted substrate

**Figure 2:**
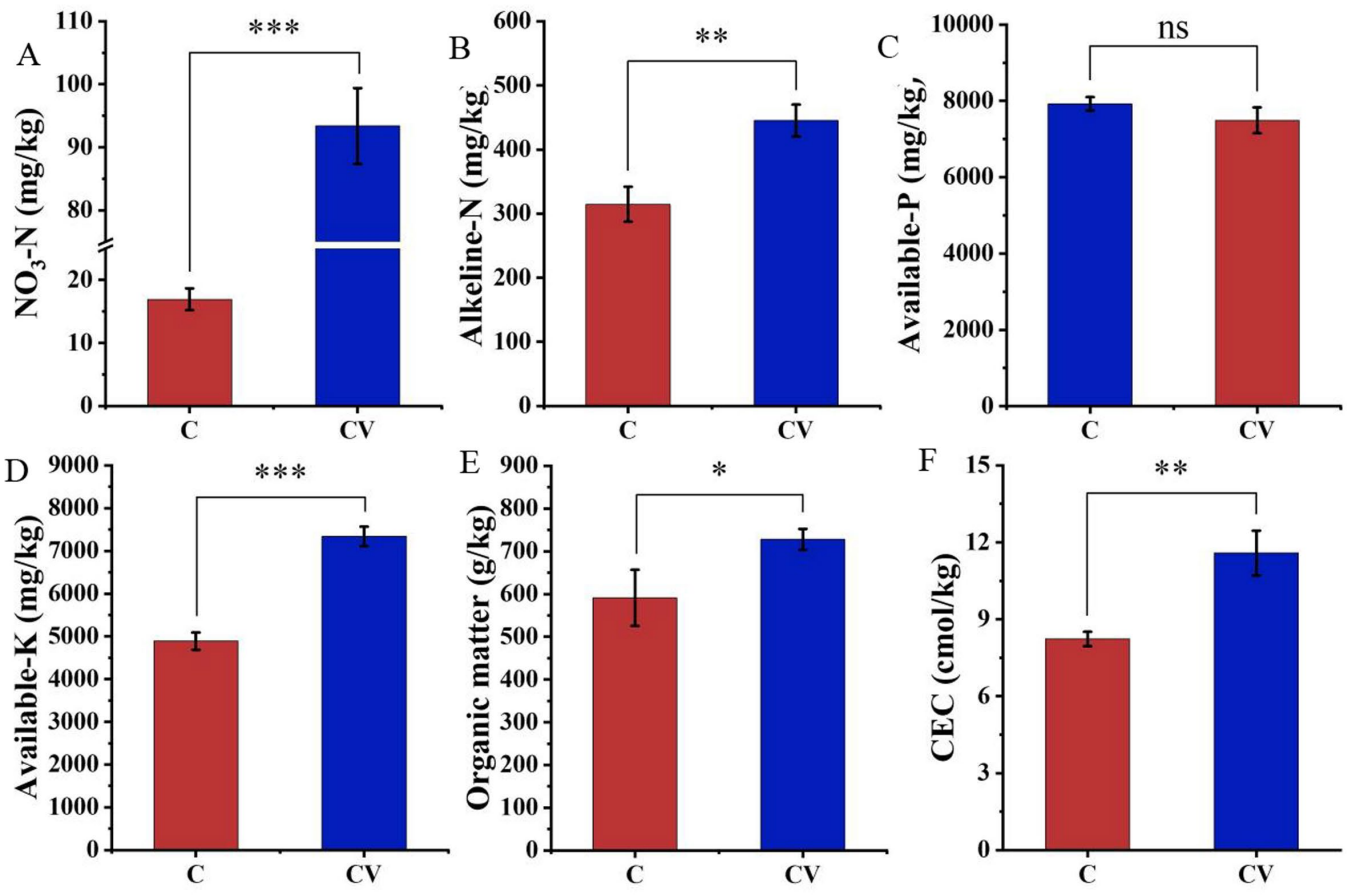
Effects of vermicompost on nutrient contents of the strawberry-planted substrate. **(A)** NO_3_-N; **(B)** alkaline-N; **(C)** available-P; **(D)** available-K; **(E)** organic matter; **(F)** cation exchange capacity (CEC). Data are mean ± SD (*n* = 15). * *p* < 0.05, ** *p* < 0.01, *** *p* < 0.001, ns: not significant.

The addition of vermicompost significantly increased the nitrogen content of the strawberry-planted substrate. For example, NO_3_-N and alkaline-N contents in the coconut coir + vermicompost treatment were 93.37 and 445.10 mg/kg, respectively, which were significantly higher than those in the coconut-coir-only treatment (16.93 and 314.60 mg/kg, respectively) (Figure 2). Meanwhile, the organic matter content, available-K, and CEC in the coconut coir + vermicompost treatment were 727.90 g/kg, 7338.87 mg/kg, and 11.58 cmol/kg, respectively, which were significantly higher than those in the coconut-coir-only treatment (590.90 g/kg, 4887.90 mg/kg, and 8.23 cmol/kg, respectively). However, vermicompost application did not have a significant effect on the available-P content of the substrate.

### 3.3 Effects of vermicompost application on strawberry plant growth

**Figure 3:**
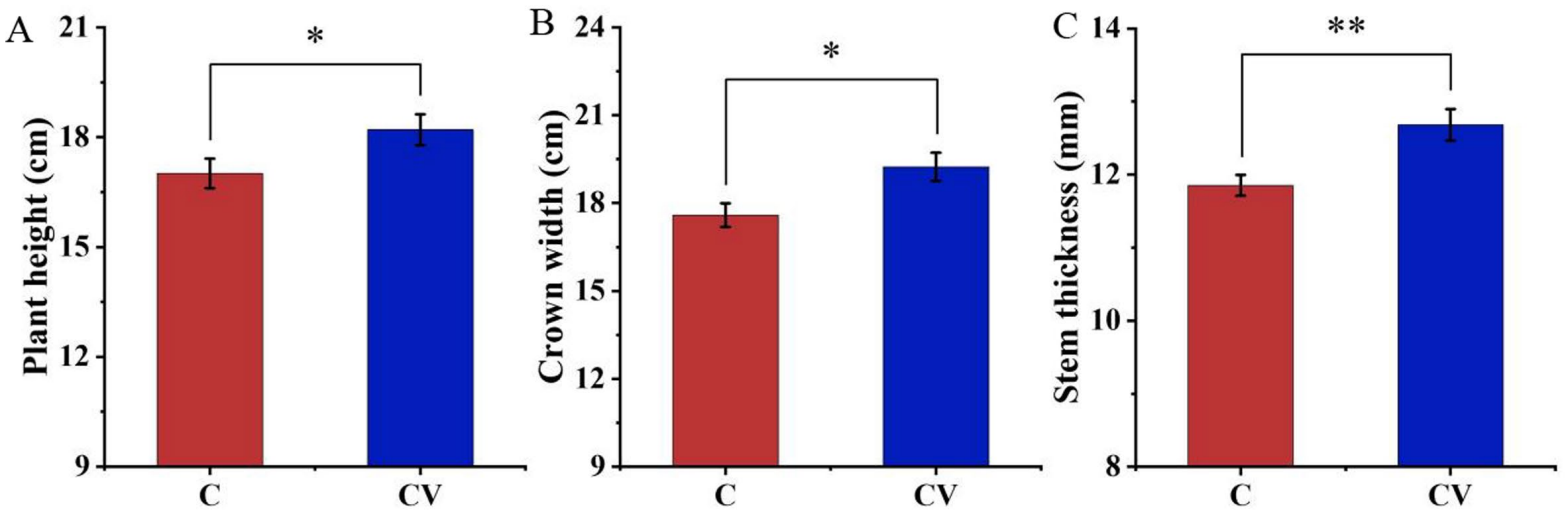
Effects of vermicompost application on strawberry plant growth. **(A)** Plant height; **(B)** crown width; **(C)** stem thickness. Data are mean±SD (*n* = 15). * *p* < 0.05, ** *p* < 0.01, *** *p* < 0.001.

**Figure 4:**
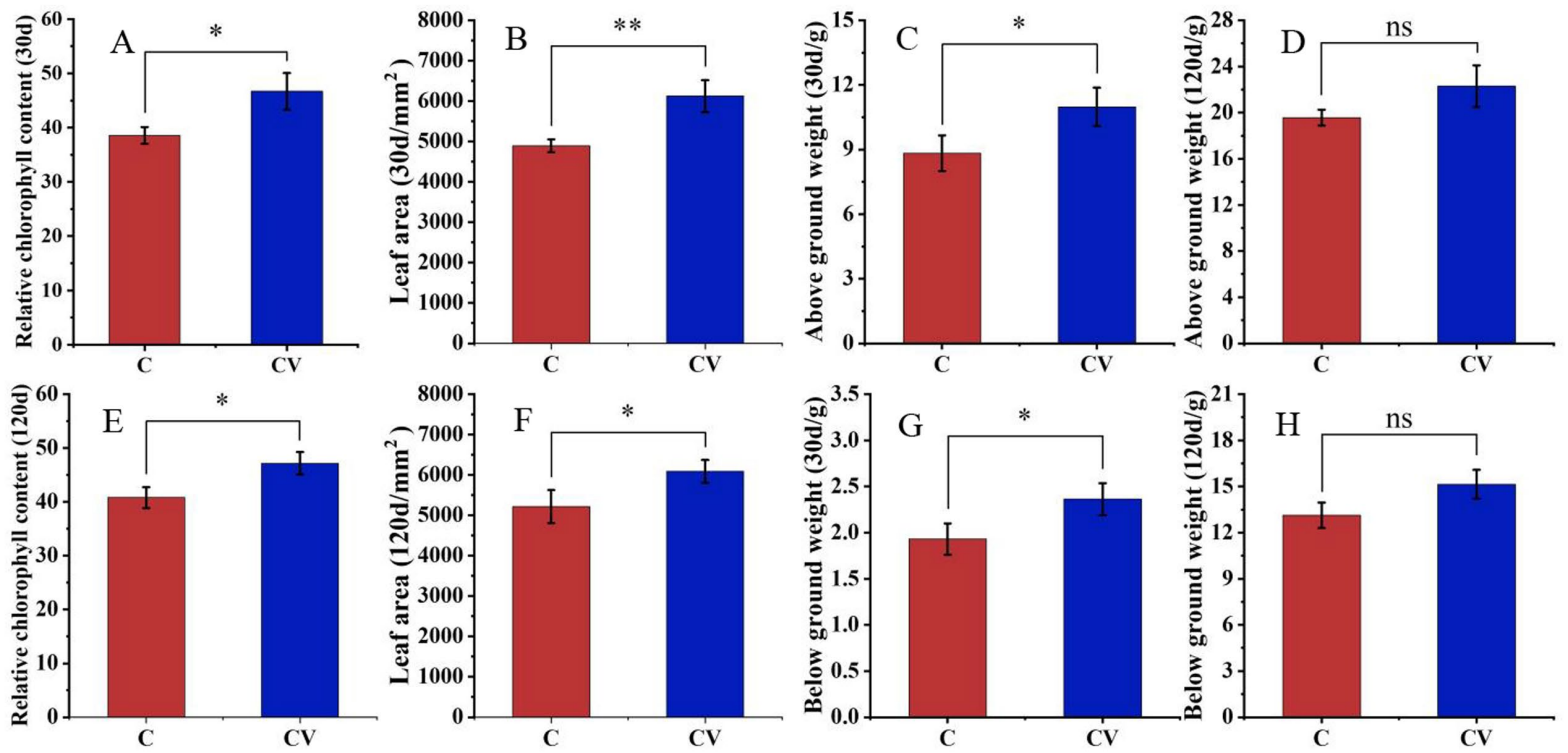
Effects of vermicompost application on strawberry plant developmental parameters 30 days after transplanting (**A**, relative chlorophyll content; **B**, leaf area; **C**, aboveground weight; **D**, belowground weight) and 120 days after transplanting (**E**, relative chlorophyll content; **F**, leaf area; **G**, aboveground weight; **H**, belowground weight). Data are mean±SD (*n* = 15). * *p* < 0.05, ** *p* < 0.01, *** *p* < 0.001, ns: not significant.

Vermicompost application effectively improved strawberry plant growth (Figures 3, 4). Forty days after transplanting, the relative chlorophyll content and leaf area of strawberry leaves in the coconut coir + vermicompost treatment were 46.68 and 6123.50 mm^2^, respectively, which were significantly higher than those in the coconut-coir-only treatment (38.56 and 4895.10 mm^2^, respectively). Meanwhile, 120 days after transplanting, the relative chlorophyll content and leaf area of strawberry leaves in the coconut coir + vermicompost treatment were 47.18 and 6088.93 mm^2^, respectively, which were significantly higher than those in the coconut-coir-only treatment (40.80 and 5215.33 mm^2^, respectively).

Thirty days after transplanting, the fresh weights of the aboveground and belowground parts of strawberry plants in the coconut coir + vermicompost treatment were 10.98 and 2.36 g, respectively, which were significantly higher than those in the coconut-coir-only treatment (8.83 and 1.93 g, respectively). With time, the difference in fresh weight between the two treatments gradually decreased; 120 days after transplanting, the fresh weights of aboveground and belowground parts did not significantly differ between the two treatments.

### 3.4 Microbial diversity of strawberry-planted substrate

**Figure 5:**
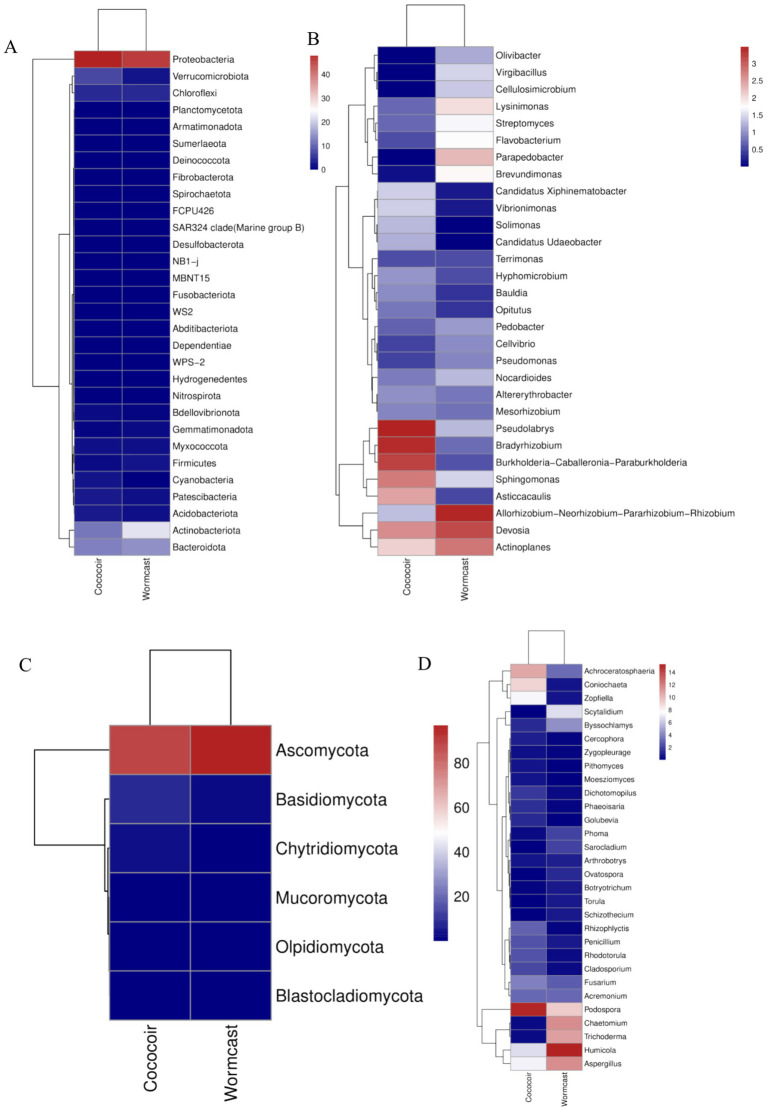
Diversity of bacterial **(A,B)** and fungal **(C,D)** communities. **(A)** Bacterial phyla; **(B)** bacterial genera; **(C)** fungal phyla; **(D)** fungal genera.

**Figure 6:**
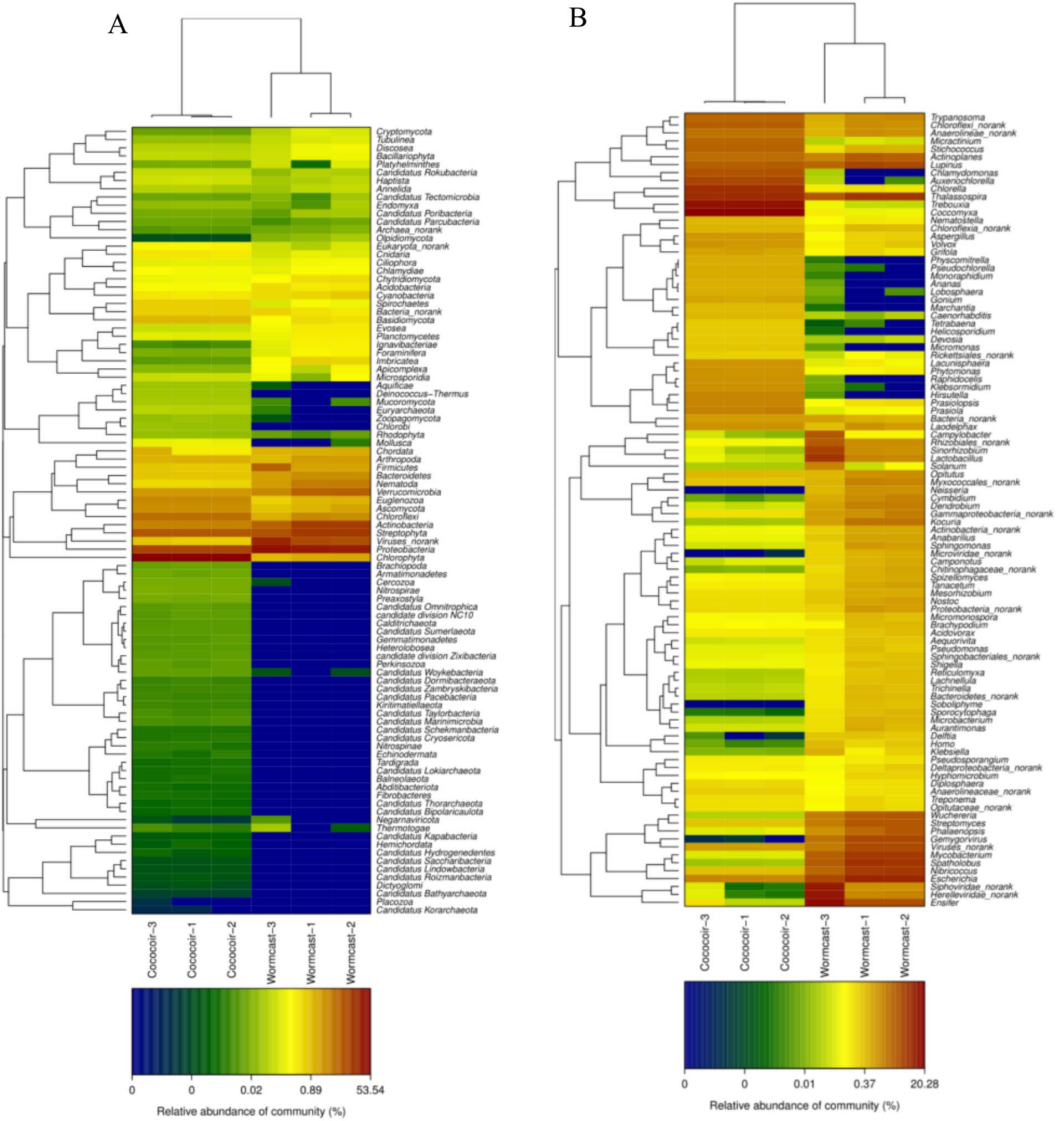
Microbial transcriptome levels of phyla **(A)** and genera **(B)** of communities.

Vermicompost application transformed the microbial diversity of the substrate (Figure 5). A total of 10 bacterial phyla (Figure 5A), the most abundant of which was *Proteobacteria*, and 42 bacterial genera (Figure 5B), the most abundant of which was *Devosia*, were detected among the bacterial communities. Among the fungal communities, five fungal phyla (Figure 5C), the most abundant of which was Ascomycota, and 30 fungal genera (Figure 5D), the most abundant of which was *Podospora*, were detected. Moreover, vermicompost application increased the beneficial microbial transcriptome level (Figures 5, 6).

### 3.5 Effects of vermicompost application on strawberry fruit and yield

**Figure 7:**
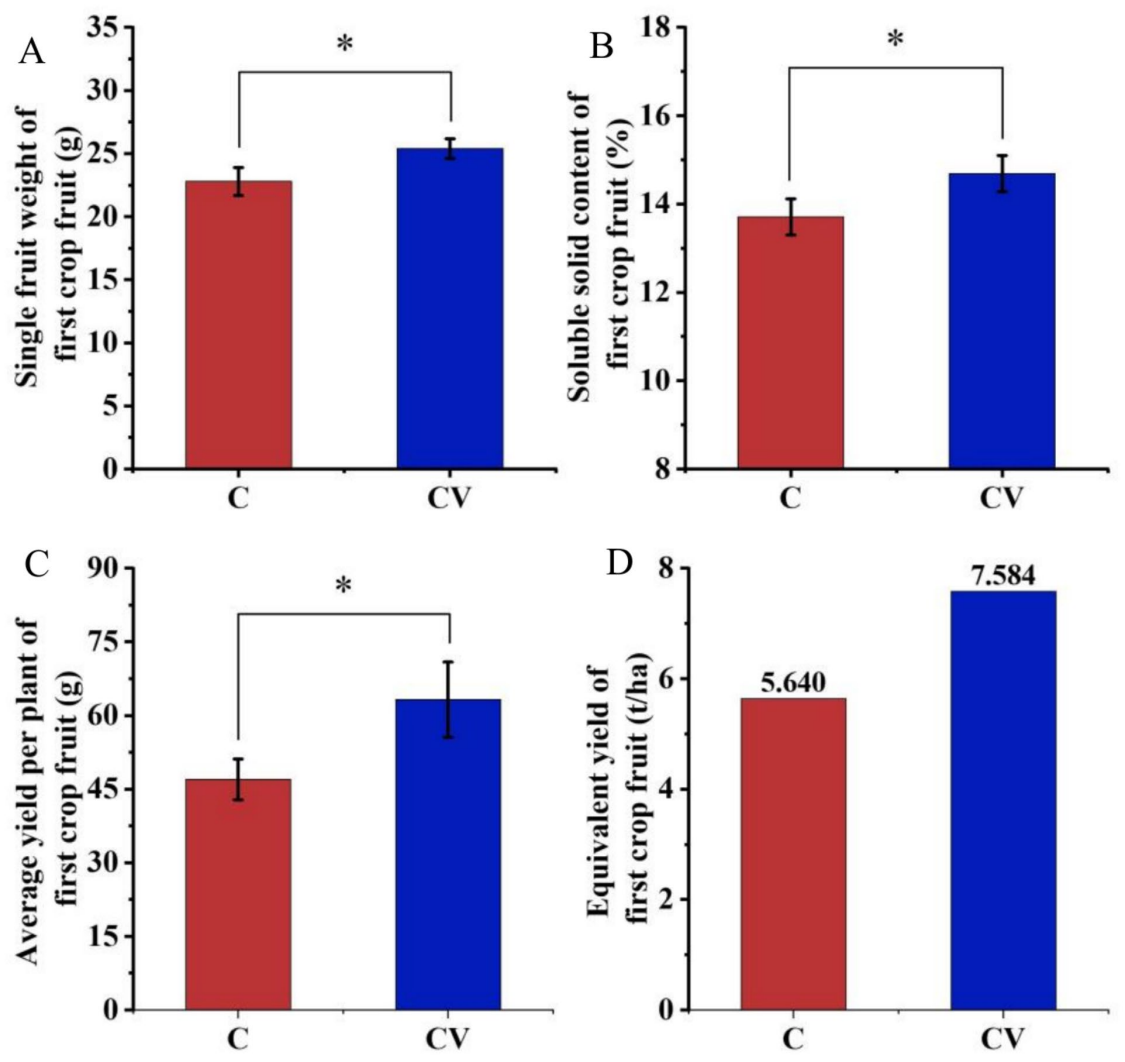
Effects of vermicompost application on strawberry fruit yield of first-crop fruit. Data are mean ± SD (*n* = 15). * *p* < 0.05, ** *p* < 0.01, *** *p* < 0.001. **(A)** Single fruit weight of first-crop fruit; **(B)** soluble solid content of first-crop fruit; **(C)** average yield per plant of first-crop fruit; **(D)** equivalent yield of first-crop fruit.

Vermicompost application advanced the average ripening time of the first strawberry crop by 7 days compared with the coconut-coir-only treatment. The single-fruit weight and soluble solid content of first-crop fruit in the coconut coir + vermicompost treatment were 25.40 g and 14.69%, respectively, which were significantly higher than those in the coconut-coir-only treatment (22.78 g and 13.71%, respectively) (Figure 7). The average plant yield of first-crop fruit in the coconut coir + vermicompost treatment was 63.20 g, which was significantly higher than the 47.00 g in the coconut-coir-only treatment. Moreover, the equivalent yield in the coconut coir + vermicompost treatment was 7.584 t/ha, which was significantly higher than the 5.640 t/ha in the coconut-coir-only treatment.

### 3.6 JNS-1 antimicrobial activity and identification

**Figure 8:**
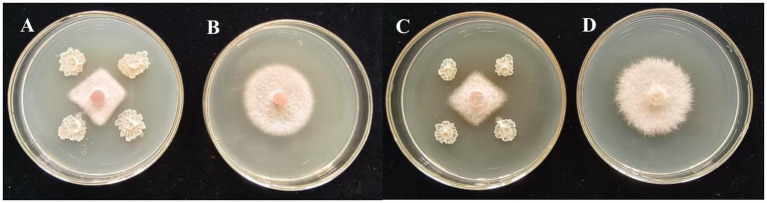
*In vitro* interaction between strain JNS-1 and the two pathogens. **(A)** JNS-1 and *Neopestalotiopsis* sp.; **(B)** *Neopestalotiopsis* sp.; **(C)** JNS-1 and *Fusarium oxysporum*; **(D)** *F. oxysporum.*

**Figure 9:**
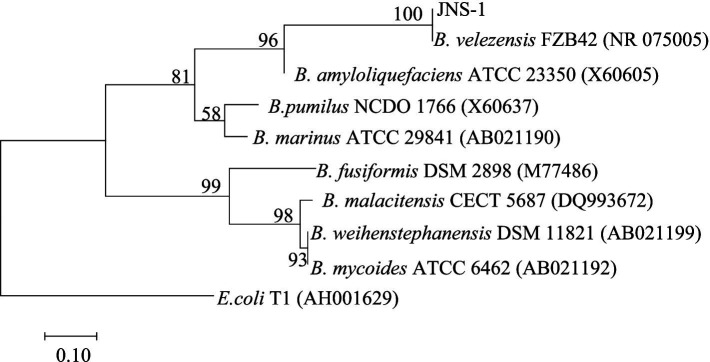
Phylogenetic tree constructed using the maximum likelihood method based on 16S rRNA regions performed in MEGA 7.0.

**Figure 10:**
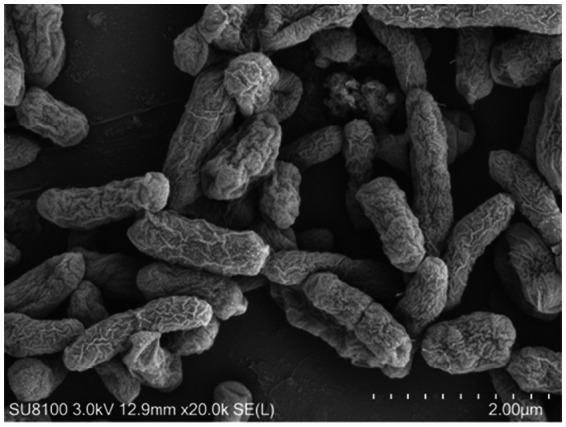
Scanning electron microscopy observation on *B. velezensis* JNS-1.

Among the 15 strains tested, JNS-1 showed the best antagonistic effect against *Fusarium oxysporum*, with an inhibitory zone rate of 59.2% (Figure 8). *B. velezensis* JNS-1 is preserved in the China General Microbiological Culture Collection Center under CGMCC no. 22531. In the 16S rRNA gene phylogenetic tree, JNS-1 (OR574161) showed a high bootstrap support value with *B. velezensis* FZB42 (NR075005), reaching 100% (Figure 9). As determined using scanning electron microscopy, *B. velezensis* JNS-1 was found to be rhabdoid and easy to aggregate to form biofilm (Figure 10).

### 3.7 Control effect in greenhouse pot experiment

According to the doses of 10 mL/plant and 20 mL/plant, the control effectiveness of JNS-1 liquid preparation on strawberry root rot disease caused by *F. oxysporum* was 80.00 and 100.00% at 15 days and 75.00% at 30 days, respectively. Furthermore, according to the dosage of 10 g/plant and 20 g/plant, the control efficiency of JNS-1 solid preparation on strawberry root rot disease caused by *F. oxysporum* was 80.00 and 90.00% at 15 days and 87.50% at 30 days, respectively.

According to the doses of 10 mL/plant and 20 mL/plant, the control effectiveness of JNS-1 liquid preparation on strawberry root rot disease caused by *Neopestalotiopsis* sp. was 66.67 and 77.78% at 15 days and 70.59 and 82.35% at 30 days, respectively. According to the dosage of 10 g/plant and 20 g/plant, the control effectiveness of JNS-1 solid preparation on strawberry root rot disease caused by *Neopestalotiopsis* sp. was 66.67 and 77.78% at 15 days and 70.59 and 82.35% at 30 days, respectively.

### 3.8 Control effect in field trials

According to the doses of 10 mL/plant and 20 mL/plant, the control effectiveness of JNS-1 liquid preparation on strawberry root rot disease at 15 days was 75.00 and 87.96%, and the control effectiveness at 30 days was 78.17 and 85.91%, respectively. According to the dosage of 10 g/plant and 20 g/plant, the control effect of JNS-1 solid preparation on strawberry root rot disease at 15 days was 76.85 and 87.04%, and the control effect at 30 days was 78.87 and 85.21%, respectively.

## 4 Discussion

Recently, in the majority of strawberry-growing regions, farmers have resorted to measures such as increased fertilizer application to maintain yields, which increases the production cost and leads to excessive environmental pollution, thereby creating a vicious circle in strawberry production (Kang et al., 2025). To ensure environmental sustainability, proper fertilization of strawberries is a crucial factor. In recent years, vermicompost has been recognized as a valuable soil conditioner for regulating plant nutrient status (Vuković et al., 2021). Moreover, the present study shows that the addition of vermicompost to coconut coir significantly increases the activity of three soil enzymes, which may regulate the metabolic balance of strawberry.

Strawberry is one of the most popular fruits in the world (Jiang et al., 2024c). However, poor soil fertility limits the growth and stunts the development of strawberry seedlings, resulting in huge economic losses (Kilic, 2023). Healthy soil plays a crucial role in increasing the crop yield, enhancing the crop’s resistance to diseases and stress, and maintaining the rhizosphere microbial community (Kang et al., 2025). In the present study, we showed that vermicompost application significantly increases the nitrogen content of the strawberry-planted substrate while also improving the strawberry plant’s growth. Nonetheless, continuous cropping can lead to an imbalance in microbial community structure in the rhizosphere of crops, which can hinder nutrient cycling and exacerbate soil acidification (Kang et al., 2025). Vermicompost can improve soil microbial functions with continuous fruit and vegetable cropping (Zhao et al., 2020). In the present study, vermicompost addition increased microbial diversity.

Moreover, our results show that vermicompost application advanced the average ripening time of the first strawberry crop by 7 days compared with the coconut-coir-only treatment. The challenges of continuous cropping in strawberry production also significantly impact both quality and yield and seriously restrict the sustainable development of the strawberry industry (Kang et al., 2025). However, due to the late development of the strawberry industry in China, a significant gap in research exists, along with numerous challenges compared to the same industry in other countries (Cao et al., 2024; Li et al., 2022). Furthermore, recycling organic waste is most important for preserving natural resources (Cabilovski et al., 2023; Manzoor et al., 2024), and fruit and vegetable quality can be improved significantly by adding recycled organic waste (Bai et al., 2024; Yeganeh et al., 2024).

Biocontrol strategies are healthy methods to control plant disease (Wang et al., 2024; Sun et al., 2022). *Bacillus* spp., including *B. velezensis* (Qiu et al., 2022), *B. subtilis* (Han et al., 2023), *B. tequilensis* (Bi et al., 2023), *B. cereus* (Yu et al., 2023), *B. amyloliquefaciens* (Xu et al., 2022), and *B. methylotrophicus* (Cheng et al., 2019), have become some of the most studied plant-growth-promoting rhizobacteria (PGPR) in recent years, and they exhibit a broad-spectrum antimicrobial activity. The biocontrol strain JNS-1 isolated from the vermicompost was identified as *B. velezensis*, and it was shown to control strawberry root rot disease in greenhouse pot experiments and field trials. In recent years, the use of *B. velezensis* has been reported to suppress various plant diseases, such as potato scab (Ma et al., 2023), tomato root rot (Chen et al., 2023), maize stalk rot (Wang et al., 2024), pepper anthracnose (Zhou et al., 2023), avocado branch blight (Li et al., 2024), and crown gall (Qin et al., 2025). Furthermore, *B. velezensis* has become one of the most studied plant growth-promoting rhizobacteria (Chen et al., 2022; Wang et al., 2024). Among *Bacillus* lipopeptides, surfactin, iturin, fengycin, and bacillomycin have been reported in many studies (Yan et al., 2021; Wang et al., 2024). The targeted application of those metabolites of *B. velezensis* JNS-1 in disease control needs to be explored in future studies.

## 5 Conclusion

The addition of vermicompost to coconut coir significantly increased the activity of three soil enzymes (cellulase, urease, and acid phosphatase); increased the growth of strawberry plants, the yield, and the nutrient content of strawberry-planted substrate; and advanced the average ripening time of the first strawberry crop. Long-term experiments might allow us to further evaluate these trends in a subsequent study. The biocontrol strain JNS-1 isolated from the vermicompost was identified as *B. velezensis.* JNS-1 showed a good direct inhibitory effect against the mycelial growth of *F. oxysporum*. The average plant yield of the first-crop fruit in the treatment that included *B. velezensis* JNS-1 was significantly higher than that in the control. It is interesting to note that, as Shandong Province has the largest strawberry cultivation area in China, the large-scale promotion of effective vermicompost-adding patterns and *B. velezensis* JNS-1 in actual strawberry production can be considered in this province in the future.

## Data Availability

The datasets presented in this study can be found in online repositories. The names of the repository/repositories and accession number(s) can be found in the article/supplementary material.
